# Wrist ganglion cyst enveloping radial artery

**DOI:** 10.12669/pjms.41.1.9776

**Published:** 2025-01

**Authors:** Jia-Fu Zhu, Kai-Chen

**Affiliations:** 1 Jia-Fu Zhu, Department of Orthopaedics, Tongde Hospital of Zhejiang Province, Hangzhou 310012, People’s Republic of China; 2 Kai-Chen, Department of Orthopaedics, Tongde Hospital of Zhejiang Province, Hangzhou 310012, People’s Republic of China

**Keywords:** Ganglion cyst, Wrist, Radial artery

## Abstract

Wrist ganglion cysts typically do not show noticeable symptoms but can affect aesthetics and even compress adjacent blood vessels and nerves. Compression therapy can eliminate cysts. However, caution is needed when treating cysts around the radial artery. This study examined a rare case of a wrist ganglion cyst that, after repeated compression and recurrence, enveloped the radial artery. This not only caused wrist swelling and pain but also increased the difficulty and risk of surgical treatment.

## INTRODUCTION

Ganglion cysts are a type of benign tumor that occur on the joint capsule and tendon sheath.^1^ Ganglion cysts typically exhibit no noticeable symptoms.^2^ However, as the cyst expands, it not only affects appearance but can also compress adjacent blood vessels and nerves, potentially causing corresponding clinical symptoms.^3^ Clinically, wrist ganglion cysts have a high incidence^4^ and commonly occur at the dorsal wrist joint capsule and the radial side of the wrist flexor tendon sheath. Compression therapy can eliminate the cyst in the short term, but the recurrence rate is high.^5^ The recurrence of ganglion cysts enveloping the radial artery is clinically rare.

## CASE REPORT

**Fig.1:**
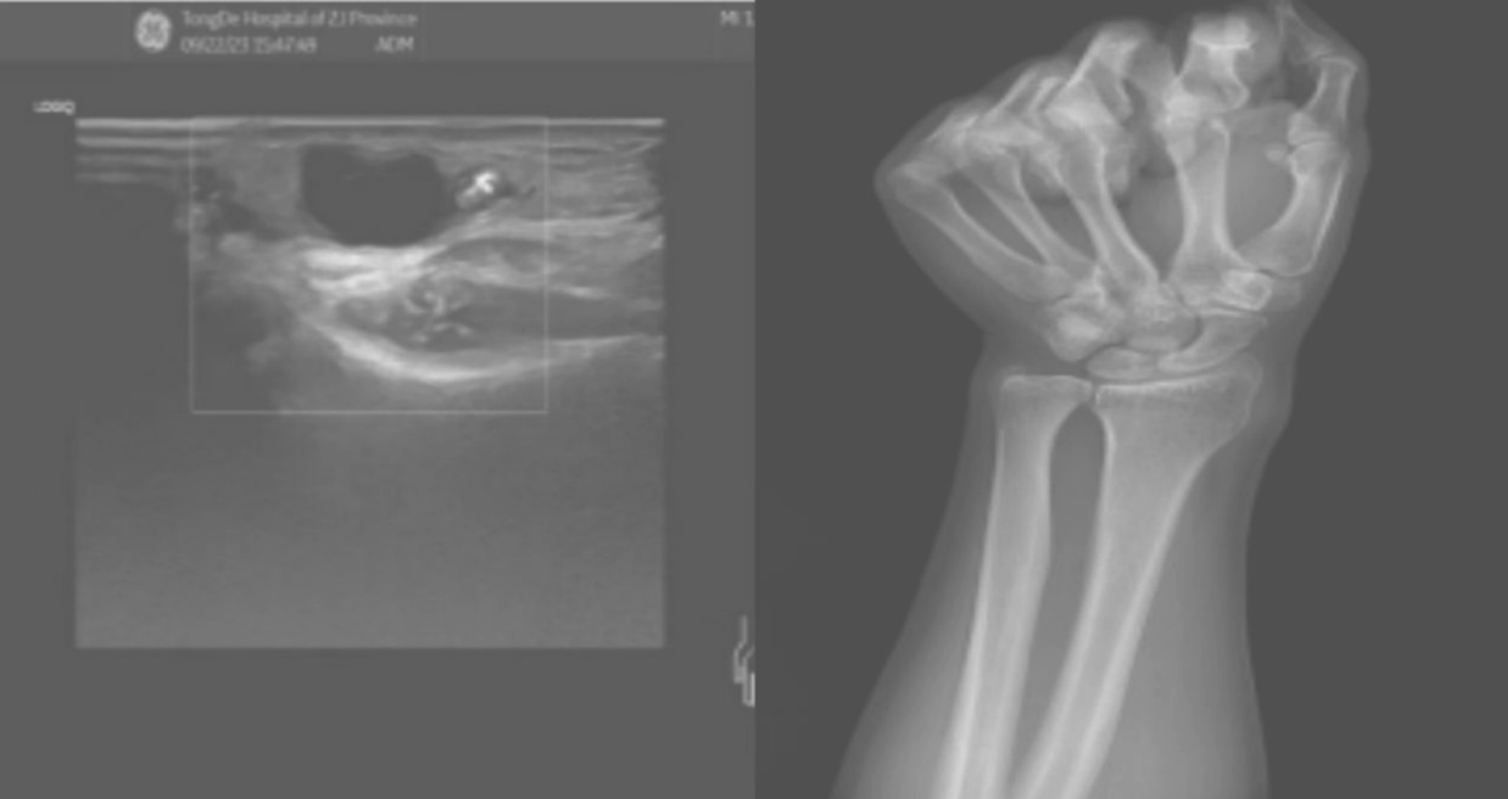
Preoperative ultrasound and X-ray.

**Fig.2:**
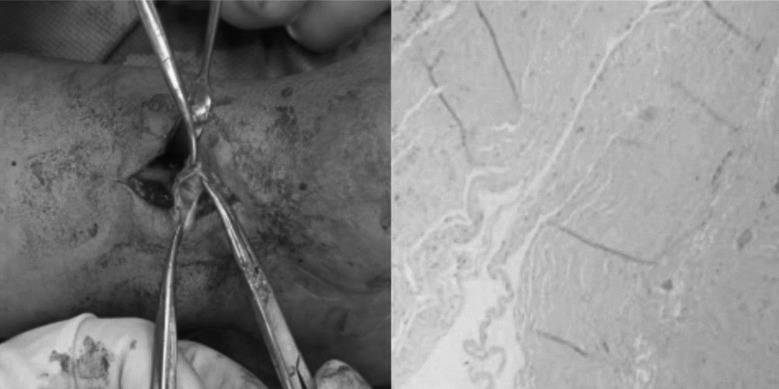
Intraoperative images and postoperative pathology.

The patient was a 67 years old man who noticed a lump on the palmar side of his left wrist 10 years ago, which was initially asymptomatic. He managed to reduce the lump by compressing it on his own, but he experienced multiple recurrences and conducted compressions over the years. Recently, the lump recurred with swelling and pain in the left wrist, and it worsened upon compression. Pre-surgical ultrasound indicated a ganglion cyst in the left wrist, and X-rays showed no bone abnormalities (Fig.1). During surgery, the cyst was separated layer by layer, and the radial artery was found entering the cyst, complicating complete removal. Opening the cyst revealed the radial artery within the cavity. The arterial pulse within the cyst was confirmed normal after releasing the tourniquet. Finally, the cyst wall and base were fully excised and sent for pathological examination. The postoperative pathological diagnosis confirmed a ganglion cyst (Fig.2).

### Ethical approval:

The study was approved by the Institutional Ethics Committee of Tongde Hospital of Zhejiang Province (No.022-JY), and written informed consent was obtained from the participant.

## DISCUSSION

The academic debate over the etiology of wrist ganglion cysts remains unresolved. One study attributed their formation to trauma and degenerative changes.^6^ Kuhlmann et al.^7^ suggested that the base of the cyst contains variable numbers of microcysts that can merge and communicate with adjacent joint spaces. Andren et al.^8^ found a one-way valve structure between the joint and ganglion cysts that prevents synovial fluid reflux, which they considered a key factor in cyst formation and enlargement. Thus, completely removing the cyst base and its one-way valve structure is crucial for reducing recurrence.

To treat wrist ganglion cysts, conservative treatments such as aspiration and compression are commonly used.^9^ Although these methods can temporarily eliminate the cyst at a low cost, the recurrence rate is high, reported between 15%–90%.^10^ The recurrence is thought to be caused by the one-way valve structure at the base of the cyst, which allows synovial fluid to enter and enlarge the cyst. In the case of the patient examined in this study, compression broke the cyst temporarily, but the one-way valve structure and microcysts remained. Repeated ruptures caused the cyst wall to envelope the radial artery, forming a rare type of ganglion cyst. The increasing fluid within the cyst not only compressed the radial artery and caused pain but also posed a high risk of arterial damage during surgery under tourniquet application, consequently complicating and risking the surgical treatment.

## CONCLUSION

Caution is needed when using compression therapy to treat ganglion cysts near major blood vessels and nerves, and surgical treatment remains the preferred method for treating ganglion cysts.

### Authors’ Contributions:

**JZ:** Designed this study, prepared this manuscript, are responsible and accountable for the accuracy and integrity of the work. **KC:** Literature search, Critical Review, Editing, revised this manuscript.

All authors have approved the final version and are accountable for the integrity of the study.
